# Regulation of Wnt/β-catenin signaling by posttranslational modifications

**DOI:** 10.1186/2045-3701-4-13

**Published:** 2014-03-04

**Authors:** Chenxi Gao, Gutian Xiao, Jing Hu

**Affiliations:** 1 Department of Pharmacology and Chemical Biology, University of Pittsburgh School of Medicine, Pittsburgh, PA 15213, USA; 2 Department of Microbiology and Molecular Genetics, University of Pittsburgh School of Medicine, Pittsburgh, PA 15213, USA; 3 University of Pittsburgh Cancer Institute, Hillman Cancer Center Research Pavilion, 5117 Centre Avenue, Pittsburgh, PA 15213, USA

**Keywords:** The Wnt/β-catenin pathway, Posttranslational modification, Phosphorylaiton, Ubiquitination, Sumoylation, Acetylation, ADP-ribosylation

## Abstract

The canonical Wnt signaling pathway (or Wnt/β-catenin pathway) plays a pivotal role in embryonic development and adult homeostasis; deregulation of the Wnt pathway contributes to the initiation and progression of human diseases including cancer. Despite its importance in human biology and disease, how regulation of the Wnt/β-catenin pathway is achieved remains largely undefined. Increasing evidence suggests that post-translational modifications (PTMs) of Wnt pathway components are essential for the activation of the Wnt/β-catenin pathway. PTMs create a highly dynamic relay system that responds to Wnt stimulation without requiring de novo protein synthesis and offer a platform for non-Wnt pathway components to be involved in the regulation of Wnt signaling, hence providing alternative opportunities for targeting the Wnt pathway. This review highlights the current status of PTM-mediated regulation of the Wnt/β-catenin pathway with a focus on factors involved in Wnt-mediated stabilization of β-catenin.

## Introduction

Wnt proteins belong to an evolutionarily conserved family of secreted cystein-rich glycoproteins. Wnts can activate β-catenin-dependent canonical Wnt pathway and β-catenin-independent non-canonical Wnt pathways, including planar cell polarity pathway and calcium pathway [1-3]. Interdisciplinary studies in the past three decades have yielded a comprehensive understanding of Wnt molecules and their downstream effects. While signaling by Wnt proteins plays pivotal roles in a wide range of developmental and physiological processes [4-8], dysregulation of Wnt pathway is linked to many human diseases including cancers [7,9,10].

**Figure 1:**
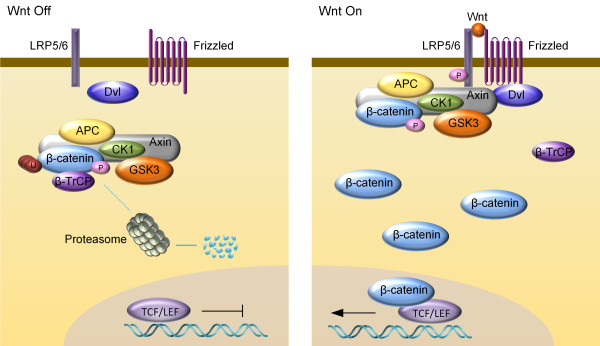
**Schematic diagram of the simplified Wnt/β-catenin pathway.** Left panel: in the absence of Wnt ligand, β-catenin is sequentially phosphorylated by CK1 and GSK3 in the cytoplasmic β-catenin destruction complex. Ubiquitin E3 ligase β-TrCP recognizes phosphorylated β-catenin and promotes its ubiquitination and proteasome degradation. Right panel: Wnt/β-catenin signaling is activated by the binding of Wnt ligand to Fz receptor and LRP5/6 coreceptors, resulting in the recruitment of Dvl and destruction complex to the membrane, which inactivates destruction complex, leading to stabilization of β-catenin. Accumulated β-catenin enters nucleus and activates target gene transcription.

A key feature of the canonical Wnt pathway is the regulated degradation of transcription coactivator β-catenin by the β-catenin destruction complex, consisting of Glycogen Synthase Kinase 3α and 3β (GSK3α and GSK3β), Casein Kinase 1 (CK1), Adenomatous Polyposis Coli (APC), scaffold protein Axin and transcription co-factor β-catenin [11]. In the absence of Wnt, β-catenin is phosphorylated by GSK3 on serine 33 and 37 and threonine 41 (which requires priming phosphorylation by CK1) [12]. Phosphorylation triggers β-catenin recruitment of ubiquitin E3 β-TrCP (β-transducin repeats-containing proteins), causing its ubiquitination and proteasomal degradation, resulting in a low level of cytoplasmic β-catenin [13,14]. Upon Wnt stimulation, Wnt ligand forms a complex with the cell-surface receptor Frizzled (Fz) and low-density lipoprotein receptor-related protein (LRP) 5/6 [4,15], and initiates a series of molecular events ultimately causing stabilization of β-catenin by suppressing phosphorylation of β-catenin [16,17] as well as β-TrCP-mediated ubiquitination and proteasomal degradation of β-catenin [18] (summarized in Figure 1). Newly synthesized β-catenin then accumulates and enters the nucleus to interact with transcription factors TCF (T-cell factor)/LEF (lymphoid enhancing factor) to activate transcription of the Wnt target genes [18].

In addition to core components of the Wnt pathway (for review, see [19,20]), non-Wnt pathway proteins also participate in the activation of Wnt signaling as regulators through modulating posttranslational modifications (PTMs) of the Wnt pathway components. By covalently adding functional groups or proteins to the target proteins, most often through enzymatic reactions, PTMs quickly change target protein’s property, relaying rapid messages in the cell, and resulting in further concerted activation of signaling cascades in response to stimuli [21]. Until now, more than 200 different types of PTM have been identified including phosphorylation, acetylation, glycosylation, methylation, ADP-ribosylation, ubiquitination and ubiquitin-like modification [22]. Besides single modifications, proteins are often modified through a combination of PTMS; different signaling pathways can be linked by PTM of shared “integrator” protein to achieve the efficient and proper cellular response. Being key mechanisms to increase proteomic diversity, PTMs are highly dynamic and largely reversible.

Most components in the Wnt/β-catenin pathway including Wnt proteins undergo one or more covalent modifications. For PTMs of Wnt proteins including glycosylation and palmitoylation, we refer the reader to two excellent reviews [23,24]. In this review, we summarize recent advances in PTM-mediated regulation of Wnt signaling with a focus on factors involved in Wnt-mediated stabilization of β-catenin and activation of β-catenin–dependent transcription (Table 1).

**Table 1:** Summary of PTMs of Wnt/β-catenin pathway components

**Protein**	**PTM**	**Sites**	**Domains**	**Involved enzymes**	**Function**	**References**
Frizzled	Phosphorylation	S576 (Xenopus Fz3)	-	-	Reduces Fz3 activity	[25]
		S554/S560 (Drosophila Fz1)	KTxxxW motif	aPKC	Inhibits Fz1 activity	[26]
	Ubiquitination	-	-	ZNRF3/RNF43	Targets for degradation	[27,28]
		-	-	UBPY/USP8 (deubiquitinase)	Targets for degradation	[29]
	Glycosylation	-	-	-	Important for Fz maturation	[30]
LRP6	Phosphorylation	T1479	Intracellular domain (ICD)	CK1γ	Recruits Axin and promotes Wnt/β-catenin signaling	[31]
		S1490	ICD	GSK3/Grk5/6/MAPKs	Recruits Axin and promotes Wnt/β-catenin signaling	[32-35]
		T1493	ICD	CK1α,γ,ϵ,δ	Recruits Axin and promotes Wnt/β-catenin signaling	[31,32]
		S1420/S1430	ICD	CK1ϵ	Suppresses LRP6-Axin interaction and β-catenin accumulation	[36]
		S1490	ICD	PKA	Essential for PT-induced β-catenin stabilization	[37]
		S1490	ICD	PFTK1/Cyclin Y	Promots Wnt/β-catenin signaling	[38]
	Palmitoylation	C1394/C1399	ICD	-	ER exit	[39]
	Ubiquitination	K1403	ICD	-	ER retention	[39]
		-	-	ZNRF3/RNF43	Targets for degradation	[28]
LRP5	Phosphorylation	PPPSPxS motifs	ICD	GSK3/CK1	Required for Axin binding	[40]
Axin	Phosphorylation	S322/S326/S330/S333/T337/ S339/T341/S343 (Rat Axin)	-	GSK3	-	[41]
		S322/S326/S330 (Rat Axin)	-	GSK3	Increases stability	[42]
		T609/S614 (Mouse Axin)	β-catenin binding domain	GSK3	Required for Axin binding to β-catenin	[43]
		S497/S500 (Mouse Axin1 isoform 2)	β-catenin binding domain	GSK3/PP1cγ (phosphatase)	Essential for Axin-β-catenin interaction	[17]
	Ubiquitination	- (K48-linked chain)	-	RNF146	Targets for degradation	[44,45]
		K789/K821 (K29-linked chain)	DIX domain	Smurf1	Disrupts Axin interaction with LRP5/6	[46]
		K505 (Mouse Axin1 isoform 1)	-	Smurf2	Targets for degradation	[47]
	Sumoylation	C-terminal KVEKVD (Mouse Axin)	DIX domain	Likely PIAS family	No effect on Wnt pathway	[48]
	ADP-ribosylation	-	-	TNKS1/TNKS2	Facilitates ubiquitin E3 binding; Promotes Wnt/β-catenin signaling	[49]
GSK3	Phosphorylation	S27 (GSK3α)/S9 (GSK3β)	-	AKT/S6K1/RSK/PKA/PKC	Suppresses kinase activity towards certain substrates	[50-54]
		Y279 (GSK3α)/Y216 (GSK3β)	Kinase domain	PYK2/GSK3	May have impact on GSK3 activity	[55-57]
		T43 (GSK3β)	-	ERK	Required for phosphorylation at Ser9 which inactivates GSK3β	[58]
		T390 (GSK3β)	-	P38 MAPK	Inactivates GSK3β	[59]
	Ubiquitination	-	-	-	Targets for degradation	[60]
	Sumoylation	K292	Kinase domain	-	Critical for kinase activity, protein stability and nuclear localization	[61]
	ADP-ribosylation	-	-	ARTD10	Inhibits activity	[62]
APC	Phosphorylation	-	-	GSK3	Increases APC binding to β-catenin	[63-65]
		S1279/S1392	-	CK1ϵ	Essential for the regulatory activity of APC towards β-catenin	[66]
	Ubiquitination	-	-	USP15 (deubiquitinase)	Targets for degradation	[67,68]
		- (K63-linked chain)		Trabid (deubiquitinase)	-	[69]
		- (K63-linked chain)	-	HectD1	Enhances APC-Axin interaction	[70]
Dvl	Phosphorylation	S139/S142 (mouse Dvl1)	-	CK1ϵ	Promotes Wnt signaling	[71]
		-	-	CK1ϵ	May enhance interaction between Dvl1 and Frat-1	[72]
		S298/S480 (Dvl2)	PDZ domain (S298)/DEP domain (S480)	RIPK4	Essential for Wnt-induced β-catenin accumulation; promotes Dvl2 signalosome assembly	[73]
		S236 (Drosophila DSH)	-	CK1ϵ	-	[74]
		-	-	PAR-1/CK2	-	[75,76]
		-	-	DDX3	May promote signalosome formation	[77]
	Ubiquitination	K413/K444/K451/K461 (Dvl1)	DEP domain	USP14 (deubiquitinase)	Suppresses Fz-Dvl interaction	[78]
		K5/K20/K34/K46/K50/K60/K69 (K63-linked chain) (Dvl1)	DIX domain	CYLD (deubiquitinase)	May enhance DVL signaling activity	[79]
		-	-	KLHL12-Cullin-3/ITCH/NEDD4L/pVHL/Malin/NEDL1	Targets for degradation	[80-85]
β-catenin	Phosphorylation	S45	-	CK1	Primes phosphorylation by GSK3	[12]
		S33/S37/T41	-	GSK3	Required for β-TrCP recognition	[12,86,87]
		S675	-	PKA	Increases stability	[88]
		S552	Armadillo (ARM) repeats domain	AKT	Promotes β-catenin disassociation from cell-cell contact and accumulation in both the cytosol and nucleus	[89]
		S191/S605	ARM repeats domain	JNK2	Critical for β-catenin nuclear localization	[90]
		T120	-	PKD1	May suppress β-catenin transcription activity	[91]
	Ubiquitination	K19/K49 (K48-linked chain)	-	β-TrCP	Targets for degradation	[14,92-96]
		- (K11/K29-linked chain)	-	EDD	Increases stability	[97]
		K394 (K63-linked chain)	-	Rad6B (ubiquitin conjugating enzyme)	Increases stability	[98,99]
		- (K11/K63-linked chain)	-	FANCL	May increase β-catenin expression and activity	[100]
		-	-	Jade-1	Targets for degradation	[101]
	Acetylation	K49	-	CBP	Inhibits β-catenin ability to activate c-myc gene	[102]
		K345	ARM repeats domain	P300	Enhances β-catenin interaction with TCF-4	[103]
		K19/K49	-	PCAF	Increases stability	[104]
TCF/LEF	Phosphorylation	T155/S166 (LEF-1)	-	Nemo-like kinase	Inhibits DNA binding of TCF/β-catenin complex	[105,106]
		T178/T189 (TCF4)				
		S154 (TCF4)	-	TNIK	Required for TCF4 transcriptional activity	[107,108]
		-	-	GSK3/CK1ϵ	Inhibits/enhances TCF3 interaction with β-catenin	[109]
		S42/S61 (LEF-1)	β-catenin binding domain	CK2	Enhances LEF-1 binding to β-catenin and transactivation	[110]
		S40 (murine LEF-1)	β-catenin binding domain	CKIδ	Disrupts LEF-1/β-catenin complex	[111]
		S147/S149/T170/S181/ T184/S190 (Xenopus TCF3)	-	HIPK2	Promotes dissociation of TCF/LEF from promoter DNA	[112,113]
		S130/T153/S164 (mouse LEF-1)				
	Acetylation	K25 (Drosophila TCF)	β-catenin binding domain	CBP	Decreases the affinity of β-catenin to TCF	[114]
		K185/K187/K188 (POP1)	-	CBP/p300	Required for POP1 nuclear localization and biological activity	[115]
		Lys150 (TCF4E2)	-	CBP	Releases inhibition by HBP1 repressor	[116]
		-	-	CBP/p300	-	[116]
	Sumoylation	K25/K267 (Mouse LEF-1)	β-catenin binding domain (K25)	PIASy	May repress LEF-1 activity by targeting LEF-1 to nuclear bodies	[117]
		K297 (TCF4)	-	PIASy, Axam	Activates β-catenin-dependent transcriptional activity of TCF4	[118]
	Ubiquitination	-	-	NARF	Targets for degradation	[119,120]

## Phosphorylation

**Figure 2:**
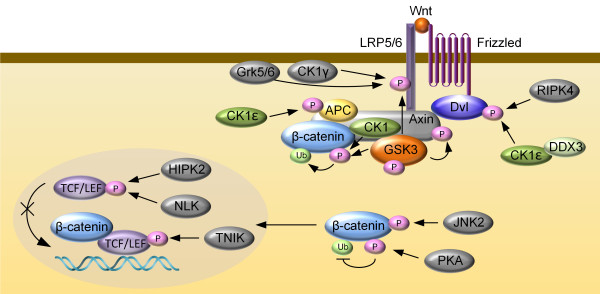
**Schematic diagram of the simplified phosphorylation-mediated regulation of the core Wnt/β-catenin pathway components.** Phosphorylation of LRP6 at T1479 by CK1γ and at S1490 by GSK3 and Grk5/6 promotes Wnt signaling. Dvl phosphorylation mediated by RIPK4 and CK1ϵ is essential for Wnt signaling. Phosphorylation of Axin at S497/S500 by GSK3 is suppressed by Wnt ligand, resulting in reduced association with LRP6 and β-catenin. C-terminal phosphorylation of β-catenin by PKA inhibits its ubiquitination and thus promotes β-catenin signaling activity. TNIK phosphorylates TCF4 to activate its transcriptional activity. NLK and HIPK2 phosphorylate TCF/LEF factors to inhibit their interaction with DNA.

Addition of a phosphate group to amino acid residues on serine, threonine or tyrosine residues, is one of the most important and well-studied post-translational modifications in eukaryotes. As one of the first PTMs to be described, phosphorylation plays critical roles in the regulation of many cellular processes; abnormal phosphorylation results in a variety of human diseases [121]. Many components of the Wnt/β-catenin pathway, including a G protein-coupled receptor proteins frizzled, Wnt co-receptor LRP6 (low density lipoprotein receptor-related protein-6), β-catenin destruction complex members (CK1, GSK3, Axin, APC, β-catenin) and disheveled (Dvl), are regulated by phosphorylation. Phosphorylation represents a key mechanism responsible for the tight control of β-catenin levels within normal cells and the activation of the Wnt/β-catenin pathway (Figure 2).

### Phosphorylation-dependent degradation of β-catenin by the β-catenin destruction complex

In the absence of Wnt, CK1α phosphorylates β-catenin at Ser45, which precedes and is required for subsequent phosphorylation of β-catenin at Ser33, Ser37 and Thr41 by GSK3 [12]. Phosphorylation of β-catenin by CK1 and GSK3 causes β-TrCP-mediated proteolysis of β-catenin, keeping the cytosolic and nuclear levels of β-catenin very low [122,123]. Upon Wnt stimulation, phosphorylation of β-catenin by GSK3 undergoes “two-phase” dynamic change: GSK3 phosphorylation of β-catenin is sharply inhibited within 30 min, phosphorylation then returns to its initial level in 2 hours [16,17] or achieve even higher level in 6 hours [17]. When normalized with respect to total β-catenin, it appears that GSK3-mediated phosphorylation of β-catenin is continuously suppressed by Wnt [12,16,17]. No significant change in CK1α-mediated phosphorylation of β-catenin is observed in 0.5-1 hour, but remarkable induction of β-catenin phosphorylation by CK1α at Ser45 is detected thereafter in different cell lines [16]. These results clearly indicate that inhibition of GSK3-medited phosphorylation of β-catenin is responsible for Wnt-induced acute stabilization of β-catenin and may contribute to Wnt-induced chronic accumulation of β-catenin. Regarding Wnt-induced long-term stabilization of β-catenin, a prior study has demonstrated that without attenuating overall GSK3-mediated β-catenin phosphorylation, Wnt abrogates β-TrCP recruitment to phosphorylated β-catenin and blocks β-catenin ubiquitination and degradation [18]. This study clearly suggests that other mechanisms are also involved in the regulation of Wnt-induced chronic stabilization of β-catenin.

Several models have been proposed to explain Wnt-mediated inhibition of β-catenin phosphorylation by GSK3: (i) Disruption of the destruction complex. Wnt induces rapid disruption of Axin/GSK3 interactions, which separates GSK3 from its substrate β-catenin, thus inhibiting β-catenin phosphorylation and causing initial stabilization of β-catenin [124]. (ii) Inhibition of GSK3 activity by LRP6. Compelling evidence indicates that Wnt-activated LRP6 can inhibit GSK3 function directly [125-128]. Results of *in vitro* and *in vivo* studies show that dually phosphorylated PPPSPxS peptides are sufficient to inhibit GSK3 kinase activity towards β-catenin and other physiological GSK3 target sites including tau and glycogen synthase [126,127]. (iii) Axin dephosphorylation. As a scaffold protein that directly interacts with other core components of the destruction complex [12,129], the scaffolding function of Axin is essential in the process of β-catenin phosphorylation by GSK3 because the interaction of GSK3β with the Axin can enhance phosphorylation of β-catenin by several orders of magnitude [130]. Axin is phosphorylated by GSK3 at Ser497/500 [17]. Upon Wnt stimulation, GSK3-mediated phosphorylation of Axin declines rapidly [17]. Dephosphorylation of Axin at Ser497/500 is carried out by PP1cγ, an isoform of PP1 catalytic subunit (PP1c) within the LRP6 signaling complex. Dephosphorylated Axin dissociates with LRP6 and β-catenin, thereby inhibiting β-catenin phosphorylation [17]. This notion is also supported by an earlier observation that phosphorylation of Axin by GSK3 increases its affinity for β-catenin [131]. Similar with this mechanism, PP1 was reported to dephosphorylates Axin at CK1-phosphorylated serine residues to reduce Axin-GSK3 interaction, contributing to β-catenin stabilization [132]. Of note, in addition to its role in β-catenin phosphorylation, phosphorylation also regulates Axin abundance: while direct phosphorylation of rat Axin on S322/S326/S330 by GSK3 stabilizes Axin [42], dephosphorylation of Axin by protein phosphatase 2C decreases the half-life of Axin [133].

GSK3β interaction with another scaffold protein APC also promotes GSK3-mediated phosphorylation of β-catenin [134]. Phosphorylation of APC by GSK3, facilitated by Axin and β-catenin and counter balanced by PP2A [63], increases APC binding affinity for β-catenin [64,65]. In addition to GSK3, APC was also reported to be phosphorylated by CK1ϵ in an Axin-dependent manner, which, in turn, confers APC’s ability to down-regulate β-catenin [66].

### Propagation of Wnt signaling through LRP6 phosphorylation

The binding of Wnt ligands to the transmembrane receptors Frizzled (Fz) and co-receptor LRP5/6 initiates a signaling cascade resulting in stabilization of β-catenin and the activation of β-catenin-dependent transcription [4,135,136]. A key step in the cascade is phosphorylation of the intracellular domain (ICD) of LRP6 at five reiterated PPPSPxS motifs and adjacent Ser/Thr cluster [31-33,137]. For a detailed summary of regulation of LRP6 by phosphorylation, we refer readers to an earlier review [138]. The enzymes catalyzing LRP6 phosphorylation have been identified: PPPSPxS motifs are sequentially phosphorylated by GSK3 (e.g., at Ser1490) and CK1 (e.g., at Thr1493) [31,32], whereas the Ser/Thr cluster (e.g., at Thr1479) is phosphorylated by casein kinase 1γ (CK1γ) [31]. Wnt-induced generation of phosphatidylinositol 4,5-bisphosphate (PtdIns(4,5)P2) at the plasma membrane is required for LRP6 phosphorylation by GSK3 and CK1γ [139]. Other key players involved in LRP6 phosphorylation have also been identified (Fz, Dvl, Axin, and PtdIns(4,5)P2) [139,140]. However, the sequence of the molecular events leading to LRP6 phosphorylation and the assembly of the LRP6 coreceptor complex remains unclear.

Different models have been proposed to depict the process: (i) Initiation and amplification of LRP6 phosphorylation [140]. In the presence of Wnt, Fz forms a complex with LRP6 and Wnt, which in turn recruits Dvl through Fz intracellular domain. Dvl directly binds to Axin [141-143], resulting in relocation of Axin and associated GSK3 to the plasma membrane to initiate LRP6 phosphorylation. The phosphorylated PPPSPxS motifs on LRP6 provide docking sites for Axin [31,137,144], leading to recruitment of additional Axin/GSK3β to form LRP6-Axin signaling complex and phosphorylate LRP6 on Ser1490 to propagate Wnt signaling [140]. (ii) LRP6 signalosome assembly [145]. Wnt induces the formation of membrane LRP6 aggregates containing Wnt pathway components, such as Fz, Dvl, Axin and GSK3 (called LRP6 signalosomes), to trigger the phosphorylation of LRP6 by GSK3 and CK1. The highly dynamic polymerization property of Dvl DIX domain, which enables Dvl self-association and co-polymerization with Axin, is important for receptor aggregation and Axin recruitment [145-147]. (iii) Wnt3a-induced PtdIns (4,5)P_2_ formation [139]. Upon Wnt stimulation, Fz transduces signal to Dvl, Dvl then directly interacts with and activates phosphatidylinositol-4-phosphate 5-kinase type I (PIP5KI). PIP5KI in turn induces PtdIns(4,5)P_2_ formation, which promotes LRP6 aggregation, LRP6 phosphorylation and Axin recruitment by unclear mechanism.

As discussed above, Dvl plays a critical role in the assembly LRP6 coreceptor complex and LRP6 phosphorylation. Dvl itself is a phosphorylation substrate: phosphorylation of Dvl can be catalyzed by RIPK4 (receptor-interacting serine/threonine-protein kinase 4), PAR-1 (Partitioning-defective 1), CK2 and CK1 [73-77]. It is known that Wnt stimulation induces Dvl phosphorylation [73,148,149], which is believed to be a critical step in Wnt signaling, however, whether Dvl phosphorylation is required for LRP6 phosphorylation or assembly of LRP6 coreceptor complex and how phosphorylation activates Dvl remain to be determined. Identification of phosphorylation sites on Dvl will help to address these questions.

### The mysterious roles of GSK3 phosphorylation in Wnt signaling

Gsk3α and Gsk3β have redundant function in the Wnt/β-catenin pathway [150]. How canonical Wnt signaling regulates Gsk3 to inhibit β-catenin proteolysis remains largely elusive. The serine/threonine protein kinase GSK3 itself is a phosphoprotein, but whether and how GSK3 phosphorylation is involved in Wnt signaling remains an open question, and evidently, contradictions exist. Catalyzed by the serine/threonine protein kinase Akt or other kinases [50-54], the N-terminus of GSK3 can be phosphorylated at Ser21 on GSK3α and Ser9 on GSK3β. Structural studies indicate that the phosphorylated N-terminus competes with the priming phosphate of GSK3 substrate for the same binding sites as a “pseudosubstrate” inhibitor, resulting in GSK3 inactivation [151]. Inhibition of GSK3 activity by Ser9/Ser21phosphorylation has been well established in the insulin pathway [53,151-153]. A prior study has shown that Wnt signaling stimulates Akt, which in turn, in association with Dvl, enhances GSK3β phosphorylation at Ser9, causing increased β-catenin level [154]. Consistent with this result, overexpression of GSK3β-Ser9A (serine mutated to alanine) abolishes insulin and IGF-1 (Insulin-like growth factor-1)-induced activation of β-catenin-dependent transcription [155]. However, the observations that GSK3β-Ser9A mutant, GSK3α-Ser21A and wild type GSK3β are regulated by Wnt signaling similarly [150,156] and that the Wnt pathway is intact in GSK3α/β^21A/21A/9A/9A^ knockin embryonic stem (ES) cells [157] appear to exclude the involvement of phosphorylation of GSK3α/β at Ser21 and Ser9 respectively in Wnt signaling.

There are several additional phosphorylation sites on GSK3 that have been reported to be associated with the regulation of β-catenin level in various biological contexts, but their role in Wnt signaling remains undetermined and elusive. Phosphorylation of GSK3β at threonine 43 by Erk (extracellular-signal-regulated kinase), primes GSK3β for phosphorylation at Ser9 by p90RSK, and mediates HBV-X protein (HBX)-induced upregulates β-catenin in human hepatocellular carcinoma cells [58]. Phosphorylation of GSK3β at threonine 390 by p38 mitogen-activated protein kinase (MAPK), which occurs primarily in the brain and thymocytes, inactivates GSK3β, leading to an accumulation of β-catenin [59]. Interestingly, Thr390 of GSK3β is not conserved in GSK3α, suggesting different regulatory mechanisms of GSK3 isoforms by phosphorylation. Consistent with the notion that p38 and phosphorylation of GSK3β at Ser9 may play a role in Wnt signaling, a prior study shows that p38 MAPK is activated upon Wnt3a stimulation and is crucial for Wnt3a-induced accumulation of β-catenin through inhibiting GSK3β a activity by inducing its phosphorylation at Ser9 [158]. Phosphorylation at tyrosine 216 in GSK3β or tyrosine 279 in GSK3α has been shown to be required for GSK3 full kinase activity using transcription factor c-Jun as an *in vitro* substrate [55,159]. In GSK3α/3β double knockout ES cells, expression wild type GSK3α reduces GSK3α/3β deficient-mediated elevation of β-catenin level, expression of GSK3α-Y279F (tyrosine replaced with phenylalanine) only partially reduces β-catenin level with respect to wild type level, likely due to impaired GSK3 kinase activity [156]. However, others have also shown that the C-terminal Tyr216 phosphorylation has no or minimal impact on GSK3 activity in *in vitro* kinase assay using myelin basic protein (MBP) or tau as substrates [160,161]. Consistent with this, it has been shown that overexpression of kinase-dead GSK3α-K148R or GSK3β–K85R remarkably enhances β-catenin-dependent transcription in the presence and absence of Wnt, whereas overexpression of GSK3α-Y279F or GSK3β–Y216F inhibits Wnt-induced activation of β-catenin-dependent transcription to a level comparable to that of WT GSK3α or GSK3β [161]. Similar to the case of kinase activity, while it has been shown that phosphorylation of GSK3β at Tyr216 impacts its binding to Axin [130,162], other evidence indicates that GSK3β with tyrosine to phenylalanine mutation at Tyr216 still retains strong binding capacity to Axin [161,163].

### Activation of β-catenin-dependent gene transcription by phosphorylation of β-catenin and TCF/LEF

In contract to the N-terminus phosphorylation by CK1 and GSK3 that triggers β-catenin ubiquitination and degradation, phosphorylation of several sites on β-catenin C-terminus (e.g., Ser675 by protein kinase A, Ser552 by AKT, and Ser191/605 by JNK2) appears to stabilize β-catenin and affect its nuclear accumulation [88-90], leading to the activation of β-catenin-dependent transcription. The TCF/LEF family proteins function as transcription repressors or activators of Wnt-responsive genes by binding to different nuclear partners, Groucho and β-catenin [164-167]. Phosphorylation of TCF/LEF family by multiple kinases has been suggested to be important for the activation of the Wnt/β-catenin pathway. The Nemo-like kinase (Nlk) family of protein kinases phosphorylates human TCF4 on two threonine residues in its central domain, Thr178 and Thr189 (and the corresponding sites Thr155 and Ser166 of human LEF-1), and inhibits the DNA binding ability of the TCF/β-catenin complex [105,106]. The kinase TNIK (Traf2 and Nck-interacting kinase,) interacts directly with both TCF4 and β-catenin and phosphorylates TCF4 to activate Wnt target gene [107,108]. Phosphorylation of human LEF-1 by CK2 at Ser42 and Ser61 increases its affinity for β-catenin and enhances gene transcription [110]. Surprisingly, however, phosphorylation of murine Ser40 residue (corresponding to human Ser42) by CKIδ disrupts the β-catenin/LEF-1 complex [111]. Both GSK3 and CK1ϵ are kinases responsible for TCF3 phosphorylation [109]. Phosphorylation of TCF3 by CK1ϵ enhances, while by GSK inhibits, TCF3 binding to β-catenin [109]. Phosphorylation of multiple members of TCF family, including LEF-1, TCF3 and TCF4, is catalyzed by homeodomain-interacting protein kinase 2 (HIPK2) [112,113]. This phosphorylation causes TCF proteins dissociation from a target promoter. Notably, HIPK2-dependent phosphorylation of transcriptional repressor TCF3 is induced by Wnt8, resulting in target gene derepression and ventroposterior development [113].

## Ubiquitination

**Figure 3:**
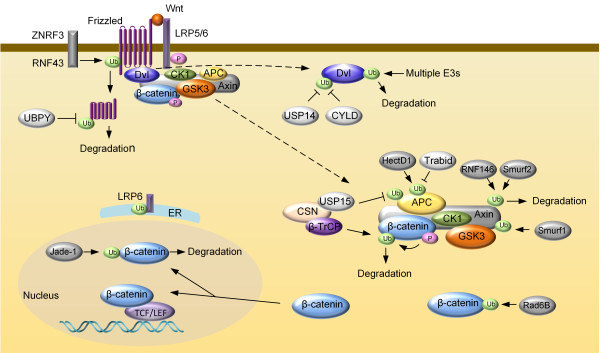
**Ubiquitination-mediated regulation of the core Wnt/β-catenin pathway components.** Cell-surface transmembrane ubiquitin E3 ligases ZNRF3 and RNF43 target frizzled for lysosome degradation. UBPY deubiquitinates frizzled to recycle it to the plasma membrane. Palmitolylation and monoubiquitylation regulate LRP6 exit from the endoplasmic reticulum (ER). Multiple ubiquitin E3 ligases target Dvl for degradation, thus negatively regulate Wnt signaling. CYLD and USP14 are deubiquitinases responsible for removing K63-linked polyubiquitin chain of Dvl. RNF146 and Smurf2-mediated ubuiqitination targets Axin for degradation, whereas Smurf1-mediated ubuiqitination of Axin regulates its interaction with LRP5/6. USP15 protects APC from degradative ubuiqitnation. HectD1 modifies APC with K63-linked polyubiquitin chain to promote interaction between APC and Axin. Apart from the β-TrCP-mediated degradative ubiquitination of β-catenin, ubiquitination-mediated by ubiquitin-conjugating enzyme Rad6B increases β-catenin stability. Ubiquitin ligase Jade-1, which is primarily localized in the nucleus, may regulate abundance of the nucleus pool of β-catenin.

Ubiquitin is an 8.5 kDa regulatory protein found in almost all tissues of eukaryotic organisms. Ubiquitination is a PTM in which an ubiquitin protein is attached to a substrate protein through an enzymatic process requiring three types of enzymes: ubiquitin-activating enzymes (E1s), ubiquitin-conjugating enzymes (E2s) and ubiquitin ligases (E3s) [168,169]. As an important PTM, ubiquitination is involved in the regulation of many basic cellular processes by regulating the degradation of proteins (via the proteasome and lysosome); coordinating the cellular localization of proteins; activating and inactivating proteins; and modulating protein-protein interactions [170-173]. These effects are mediated by different types of substrate ubiquitination: adding one ubiquitin molecule to one substrate lysine residue (monoubiquitination) or several lysine residues (multi-monoubiquitination); adding an ubiquitin chain on a single lysine residue on the substrate protein (polyubiquitination) [174]. Polyubiquitin chains are built by the formation of an isopeptide bond between Gly76 of one ubiquitin to the epsilon-NH2 group of one of the seven potential lysines (K6, K11, K27, K29, K33, K48 or K63) of the preceding ubiquitin [175,176]. A special polyubiquitination chain, the head-to-tail linear polyubiquitin chain, is formed by linking the N-terminal amino group of methionine on the ubiquitin conjugated with a substrate protein and the C-terminal carboxyl group of the incoming ubiquitin [177,178]. The various types of ubiquitination are linked to distinct physiological functions in cells. While lysine 48-linked chains target proteins for degradation [173]; other types of ubiquitin linkages mediates proteolytic as well as non-proteolytic functions including endocytic trafficking, lysosomal turnover and DNA repair [175,179,180]. Like phosphorylation, ubiquitin modification of Wnt pathway proteins has emerged as a key mechanism that determines the pathway activity (Figure 3).

### Regulation of turnover (proteasomal and lysosomal degradation) of β-catenin, Axin, APC and Dvl by ubiquitin

Modulation of the abundance of the Wnt pathway components through ubiquitination-mediated proteasomal and lysosomal degradation plays a critical role in the regulation of Wnt signaling. A characteristic feature of the canonical Wnt pathway is tight regulation of the level of β-catenin controlled by CK1- and GSK3β-mediated phosphorylation and subsequent proteolytic degradation. In the absence of Wnt, phosphorylation of the N-terminus of β-catenin—Ser45 by CK1α, followed by GSK3-mediated phosphorylation of Ser33, Ser37 and Thr41— triggers the recruitment of the β-TrCP [12,14,86,123], the substrate-recognition subunit of a multi-protein Skp1-Cullin-F-box (SCF) RING-type E3 ligase [181]. The SCF^β-TrCP^-ubiquitin ligase complex subsequently attaches K48-linked polyubiquitin chains onto lysine residues 19 and 49 at the N-terminus of β-catenin [92,93], causing its proteasomal degradation. β-TrCP recruitment to and ubiquitination of β-catenin is inhibited upon Wnt stimulation despite of phosphorylation of β-catenin by CK1 and GSK3 [18]. How this regulation is achieved remains an open question. The plant homeodomain protein (PHD) Jade-1 is also found to mediate β-catenin ubiquitination and degradation [101]. Like β-TrCP, Jade-1 directly interacts with the N-terminus of β-catenin in a phosphorylation-dependent manner. However, unlike β-TrCP which only ubiquitylates phosphorylated β-catenin, Jade-1 ubiquitylates both phosphorylated and non-phosphorylated β-catenin and therefore regulates canonical Wnt signaling in both Wnt-off and Wnt-on phases. Since Jade-1 is primarily localized in the nucleus [182,183], it may mainly regulate the nuclear pool of β-catenin, whereas β-TrCP is responsible to degrade cytoplasmic β-catenin [94]. This may explain why Jade-1 silencing cannot be completely compensated for by β-TrCP [101]. The stability of Jade-1 is dependent on the presence of a functional von Hippel-Lindau (VHL) protein [183], downregulation of Jade-1by VHL mutations is thought to be responsible for the hyperactivation of the Wnt pathway in renal cell carcinoma. In contrast to ubiquitination by β-TrCP and Jade-1, ubiquitination of β-catenin by the E2 ubiquitin conjugating enzyme Rad6B and the E3 ubiquitin ligase EDD stabilizes β-catenin [97-99]. Both Rad6B and EDD interact with β-catenin and promote its ubiquitination, leading to increased level and enhanced activity of β-catenin. EDD promotes the attachment of K29-linked and/or K11-linked polyubiquitin chains to β-catenin [97], whereas Rad6B adds K63-linked polyubiquitin chain to Lys 394 of β-catenin [98,99], suggesting that Rad6B (E2) may not couple with EDD (E3) to attach polyubiquitin chain to β-catenin. Together, it appears that different ubiquitin E2 or E3 may attach different types of ubiquitin chain to different lysine residues on β-catenin, causing context-specific functional consequences.

Axin (Axin1 and Axin2) is a scaffold protein that directly interacts with other core components of the destruction complex [129,184,185]. Being the rate-limiting factor of the destruction complex, Axin abundance is a determinant factor for the assembly of the multi-protein destruction complex [186,187], and the cellular level of Axin is tightly controlled. Wnt induces polyubiquitination-mediated proteasome degradation of Axin, an event that is believed to impair the formation of sufficient destruction complex, facilitating Wnt-induced β-catenin stabilization. A recent study has revealed that the poly-ADP-ribosylation of Axin catalyzed by poly-ADP-ribosylating enzymes tankyrase (TNKS) 1 and tankyrase 2 is a prerequisite for Axin ubiquitination [49]. Until now, two ubiquitin E3 ligases have been implicated in Axin ubiquitination and degradation: the smad ubiquitination regulatory factor 2 (Smurf2) [47] and the RNF146 RING-type ubiquitin E3 ligase [44,45]. Smurf2 directly interacts with Axin and specifically ubiquitylates lysine 505 on Axin [47]. RNF146 binds to and ubiquitinates poly-ADP-ribosylated Axin for degradation to promote Wnt signaling [44,45]. The ubiquitination-mediated degradation of Axin is counterbalanced by the ubiquitin protease USP34 [188]. Whether and how the activities of these E3 ubiquitin ligases (Smurf2 and RNF146) and the deubiquitinating enzyme USP34 are regulated upon Wnt simulation is currently unknown. Furthermore, how these ubiquitin ligases cooperate to share their responsibility for ubiquilating Axin remains to be determined. For example, do Smurf2 and RNF146 ubiquitinate Axin in different pools or different complexes? Do they couple with different E2 ubiquitin conjugating enzymes to ubiquitinate different lysine residues on Axin? Answers to these questions will help to elucidate molecular mechanism underlying Axin regulation. Nevertheless, given that small-molecule inhibitor of TNKS1 and TNKS2 exerts anti-tumor effect through downregulating Wnt signaling by function as an Axin stabilizer [49], Poly-ADP-ribosylation -dependent ubiquitination of Axin provides an alternative and promising opportunity for targeting Wnt pathway for cancer therapy.

Considered as a Wnt pathway negative regulator, the scaffold protein APC facilitates GSK3-mediated phosphorylation of β-catenin [134]. APC is also an ubiquitin substrate. Ubiquitination of APC, which targets APC for proteasome degradation, is facilitated by Axin and is suppressed by Wnt3a [67]. The responsible E3 ubiquitin ligase is currently unknown. The deubiquitinase USP15 (Ub-specific protease 15) has been implicated in the ubiquitination-mediated degradation of APC [68]. USP15 is a key component of the COP9 signalosome (CSN), which regulates the ubiquitin proteasome system (UPS) by controlling cullin-RING Ub ligases [189]. The CSN complex associates with the SCF^β-TrCP^ E3 complex to form a supercomplex. The CSN supercomplex regulates the balance between β-catenin and APC: while it stimulates β-catenin degradation, USP15 associated with the CSN stabilizes APC. Upon Wnt stimulation, the CSN complex dissociates from SCF^β-TrCP^ and the APC-Axin complexes, rendering APC susceptible for proteolysis. This model suggests that Wnt-induced degradation of APC promotes β-catenin stabilization, which is not consistent with earlier studies showing stabilization of APC upon Wnt signaling [67,190].

Dvl (three vertebrate isoforms: Dvl1, Dvl2 and Dvl3) is the decision point for downstream canonical and non-canonical Wnt signaling branches and plays a critical role in the relay of signals from the LRP6 receptor complex to downstream effectors in the Wnt/β-catenin pathway [191-194]. The level of Dvl is tightly regulated by ubiquitination-mediated degradation. Several ubiquitin ligases have been identified as negative regulator of Wnt signaling by physically interacting with Dvl to enhance its ubiquitination and subsequent degradation through proteasome or lysosome under different physiological conditions [80-84]. In a Wnt-dependent manner, the BTB-protein Kelch-like 12 (KLHL12) binds to Dvl, promoting its poly-ubiquitination and degradation and antagonizing the Wnt–β-catenin pathway in cultured cells, Xenopus and zebrafish embryos [80]. Wnt stimulation is known to hyperphosphorylates Dvl, which is required for the full activation of the Wnt pathway [71,73]. The HECT-containing Nedd4-like ubiquitin E3 ligase ITCH interacts with Dvl, the interaction requires both the PPXY motif and the DEP domain of Dvl. ITCH ubiquitinates and degrades phosphorylated Dvl and but does not appear to influence the function of nuclear Dvl in the Wnt signaling pathway [81].

NEDD4L (neural precursor cell expressed, developmentally down-regulated 4-like) is a member of the NEDD4 family ubiquitin ligases, directly binds to Dvl2 through the WW3 domain of NEDD4L and the PY motif of Dvl2, and targets Dvl2 for proteasomal degradation through K6-, K27-, and K29-linked atypical ubiquitination [82]. By promoting Dvl2 degradation, NEDD4L negatively regulates the Wnt/β-catenin pathway and antagonized Dvl2-induced axis duplication in Xenopus embryos. In a recent study autophagy was shown to negatively regulate Wnt signaling by promoting Dvl degradation through Von Hippel–Lindau protein (VHL)-mediated polyubiquitination [83]. VHL, a component of an SCF (Skp1–Cdc53–F-box)-like ubiquitin E3 ligase complex, binds to Dvl through Dvl’s DEP domain and ubiquitinates Dvl, ultimately causing Dvl degradation through the autophagy–lysosome pathway. The negative association of Dvl protein with autophagy in human colon cancer suggests a clinical relevance of this finding [83]. The RING finger domain containing ubiquitin E3 ligase Malin is also found to interact with Dvl2 and promote polyubiquitination of Dvl through K48- and K63-linked ubiquitin chains, leading to its degradation through both proteasome and autophagy [84].

The involvement of multiple ligases highlights the importance of tight control of Dvl level in cells. But how these E3 ubiquitin ligases—KLHL12, ITCH, NEDD4L, VHL and Malin—distinguish themselves from each other as an ubiquitin ligase for Dvls remains unclear. It is possible that they act on Dvl in a different format or different isoform of Dvl. For example, ITCH only ubiquitinates phosphorylated Dvl, whereas pVHL and KLHL12 ubiquitinates both phosphorylated and unphosphorylated Dvl, and KLHL12 seemed to prefer targeting Dvl3 over Dvl2 [80]. It is also possible that these ligases are regulated differently in response to stimuli including Wnt in a tissue-specific manner or within specific subcellular compartments.

### Regulation of Wnt receptor LRP6 and Fz trafficking by ubiquitin

The levels of Wnt receptor LRP6 and Fz, not surprisingly, greatly impact the activation of the pathway [195,196]. The membrane level of LRP6 is largely regulated by the interplay between PTMs: palmitoylation, a covalent attachment of fatty acids to cysteine and less frequently to serine and threonine residues of proteins, and ubiquitination [39]. LRP6 is palmitoylated shortly after synthesis and remains palmitoylated, which is required for LRP6 exits from endoplasmic reticulum (ER). Without palmitoylation, LRP6 is retained in the ER due to monoubiquitination on lysine 1403. Mutation of this site leads to a full recovery of membrane targeting of palmitoylation-deficient LRP6. Notably, the responsible ubiquitin E3 ligase and deubiquitinating enzyme for LRP6 ubiquitination, and the types of ubiquitin chains have not been revealed.

Ubiquitination commonly drives cell-surface receptor internalization and lysosomal degradation [197]. The ubiquitination of Fz is mediated by cell-surface transmembrane E3 ubiquitin ligase zinc and ring finger 3 (ZNRF3) and its homologue ring finger 43 (RNF43) [27,28]. As a negative regulator of the Wnt pathway, RNF43 interacts with FZD5, promotes its ubiquitin-mediated endocytosis, thereby negatively regulating Wnt/β-catenin signaling [27]. ZNRF3, another ubiquitin E3 ligase for Fz, forms a receptor complex with R-spondin proteins [28]. Without R-spondin, ZNRF3 ubiquitylates frizzled and promotes its degradation, therefore inhibiting Wnt signaling. When R-spondin is present, it clears out ZNRF3 from the membrane by inducing the interaction between ZNRF3 and the stem-cell marker LGR4, resulting in accumulation of frizzled and LRP6 on the plasma membrane and enhancing Wnt signaling. In contrast to ubiquitination-mediated lysosomal trafficking and degradation, de-ubiquitination controls the recycling of receptors, including frizzled receptors [29]. Fz is stabilized by UBPY/Ub-specific protease 8 (USP8)-mediated deubiquitination, which leads to led to recycling of Frizzled to the plasma membrane, thereby upregulating Wnt signaling [29].

### The nonproteolytic roles of K63-linked polyubiquitination of APC and Dvl and K29-linked polyubiquitination of Axin

The most studied function of ubiquitination in the Wnt pathway relates to protein turnover. However, emerging evidence indicates that nonproteolytic function of polyubiquitination of core Wnt pathways proteins through lysine 63 plays an important role in the regulation of the pathway [69,70,79,198]. In 2008, Tran et al. reported the first direct evidence indicating a role of K63-linked ubiquitin chain during Wnt-induced transcription [69]. Trabid, a DUB enzyme, is found to preferentially binds to K63-linked ubiquitin chains with its three tandem NZF fingers (Npl4 zinc finger), and to cleaves these chains with its OTU (ovarian tumor) domain. Trabid binds to and deubiquitylates APC. Although Trabid targets APC ubiquitination and function as a positive regulator of Wnt signaling in mammalian and *Drosophila* cells, surprisingly, it acts below the stabilization of β-catenin. How Trabid-mediated deubiquitination of APC links to the activity of the TCF–β-catenin transcription complex remains unsolved. Two later studies shed more light on the molecular mechanisms underlying K63-linked ubiquitination of APC in Wnt signaling [70,198]. APC is modified predominantly with K63-linked ubiquitin chains when it is bound to Axin in unstimulated HEK293 cells, which requires a fully assembled APC/Axin/GSK3β/phospho-β-catenin complex [198]. Wnt3a stimulation inhibits K63-linked ubiquitination of APC in a time-dependent manner, an event coincides with the disassociation of Axin from APC and the stabilization of cytosolic β-catenin, indicating that K63-linked polyubiquitination of APC impacts the assembly and/or efficiency of the β-catenin destruction complex. This finding was further confirmed by the observation that the E3 ubiquitin ligase HectD1 modifies APC with K63-lined polyubiquitination and promotes the APC/Axin interaction to negatively regulate Wnt signaling [70]. Together, these studies have established a negative correlation between K63-linked ubiquitination of APC and activation of the Wnt pathway. Future identification of the ubiquitination site(s) in APC will enable mutational analysis and more conclusive determination of the importance of K63-lined ubiquitination of APC in Wnt signaling and other APC-regulated cellular processes.

Both positive and negative roles of K63-lined ubiquitination of Dvl in the Wnt regulation have been reported [78,79]. A prior study shows that the N-terminus of Dvl1 (K5, 20, 34, 46, 50, 60 and 69 on the DIX domain) is modified by K63-linked polyubiquitination, which requires DIX-domain- mediated polymerization of Dvl [79]. The deubiquitinating enzyme CYLD binds to and deubiquinates Dvl, inhibiting the signaling activity of Dvl and the activation of the Wnt pathway. CYLD is a familial cylindromatosis tumor suppressor gene, mutations in the CYLD gene cause human familial cylindromatosis [199]. The finding that hyperactive Wnt signaling in human cylindroma skin tumors arises from mutations in CYLD validates the clinical significance of CYLD-mediated deubiquitination of Dvl [79]. In contrast to the positive role of K63-linked ubiquitination of Dvl in Wnt signaling [79], a recent study shows that K63-linked ubiquitination of Dvl on four lysines at the C-terminus within the DEP domain (K413, 444, 451 and 461) plays a negative role in the regulation of Wnt signaling [78]. Usp14, identified as a deubiquitinase for Dvl, transiently interacts with Usp14 upon Wnt stimulation, disassembles K63-linked polyubiquitin chains attached to Dvl, an event that is required for Wnt signaling. Tissue microarray analysis of colon cancer has revealed a strong correlation between the levels of Usp14 and β-catenin, providing further support for the negative of K63-linked ubiquitination of Dvl in Wnt signaling. Together, these two studies suggest that ubiquitination of Dvl on lysines clustered in different domains (i.e., the N-terminal DIX domain vs. the C-terminal DEP domain) through K63-lined ubiquitin chains involves different deubiquitinase and plays seemingly contradictory roles (positive and negative) in Wnt signaling. Since both studies used HEK293 cells as a cellular model for most of the mechanistic experiments [78,79], the different outcome of Dvl ubiquitination appears not to result from cell-type-specific or cellular context-specific effects. The contradictory roles of K63-linked ubiquitination of Dvl add further complexity to the understanding of the molecular mechanisms underlying the still perplexing role of Dvl in Wnt signaling.

Ubiquitination of Axin by ubiquitin ligases Smurf2 and RNF146 mediates Axin degradation [44,45,47]. Smurf1 is recently identified as an additional ubiquitin E3 ligase for Axin ubiquitination [46]. Unexpectedly, smurf1-mediated Axin polyubiquitination at Lys789/821 with K29-linked polyubiquitin chain does not cause its degradation, but instead disrupts its association with LRP5/6, resulting in suppression of LRP6 phosphorylation at Ser1490. Consistent with its negative role in Wnt signaling, Axin ubiquitination with K29-linked polyubiquitin chain is significantly suppressed by Wnt3a stimulation.

## Sumoylation

In addition to ubiquitin, there is a growing family of ubiquitin-like proteins (UBLs) that modify cellular targets in a pathway parallel to, but distinct from, that of ubiquitin. SUMO (Small Ubiquitin-like Modifier) proteins are a family of small proteins that are around 100 amino acids in length and 12 kDa in mass. Mammalian SUMO has four isoforms: SUMO1, SUMO2, SUMO3, and SUMO4. SUMO2 and SUMO3 share 95% sequence homology and are distinct from SUMO-1(often collectively referred to as SUMO2/3). Sumoylation refers to the process that small ubiquitin-related modifier (SUMO) is covalently attached to the target proteins through sequential enzymatic reactions involving the activity of SUMO activating enzyme (E1), SUMO conjugating enzyme (E2) and SUMO ligase (E3) [200,201]. Sumoylation is a reversible process. Removal of SUMO from its target proteins is carried out by members of SUMO-specific proteases (SENP) family [202]. SUMO was identified as a post-translational modifier almost two decades ago [203], and our knowledge of sumoylation has been greatly expanded since then. We now know that at any given time, only a small portion of a particular substrate is modified by SUMO, but the very small amount of sumoylated proteins play an important role in the regulation of diverse signaling pathways and is critical to maintain normal cell function [204,205].

The identification of Axam (Axin Associating Molecule) as a novel Axin-binding protein and a negative regulator of Wnt/β-catenin pathway provides the first evidence that sumoylation is involved in the regulation of Wnt signaling [206]. Axam belongs to the SENP family, and downregulation of β-catenin by Axam requires its DeSUMOylation activity [207]. Later, sumoylation of LEF1 is found to negatively regulate LEF1 transcriptional activity by sequestration into nuclear bodies [117]. The protein inhibitor of activated gamma (PIASy) is identified as a LEF1 SUMO E3 ligase [117]. In contrast, PIASy-dependent sumoylation of TCF4, another member of TCF/LEF family, appears to activate β-catenin-dependent transcriptional activity of TCF4, whereas Axam has opposing effect [118].

Sumoylation of the scaffold protein Axin at its C-terminal six amino acids stretch (C6 motif), a motif that is critical for Axin interaction with three SUMO-1 E3s, PIAS1, PIASxβ and PIASy, prevents its polyubiquitination, thus increasing its stability [48,208]. Given the fact that Axin is the concentration-limiting component in the β-catenin destruction complex [49,186], it is reasonable to expect that sumoylation of Axin is involved in the regulation of Wnt signaling by controlling Axin steady-state level. Surprisingly, it appears not to be the case because wt Axin and Axin sumoylation-defective mutants destabilize β-catenin and inhibit LEF1 transcriptional activity at similar level [48]. GSK3β is found to be sumoylated at lysine 292 [61]. Mutation of the lysine 292 inhibits GSK3β activity toward tau and reduces GSK3β stability. However, whether sumoylation of K292 on GSK3β plays a role in Wnt signaling remains unexplored. Transducin β-like protein 1 (TBL1) and TBL1-related protein (TBLR1) function as transcriptional coactivators in canonical Wnt pathway by recruiting β-catenin to the Wnt target gene promoters [209]. A prior study indicates that Wnt-dependent sumoylation of TBL1 and TBLR1 releases them from SMRT/N-CoR corepressor complex and enhances the formation of the TBLR1-TBL1-β-catenin complex and their recruitment to Wnt target gene promoters [210]. Sumoylation or ubiquitination fusion protein methodology is a powerful complementary strategy for mutant approach that is used to discriminate between the consequence of lacking substrate sumoylation and conformational change-related artifacts [211-215]. The observation that fusion SUMO1 to sumoylation mutants of TBL1 and TBLR1 restores the activity of TBL1 and TBLR1 thus validates the functional role of sumoylation in the regulation of TBL1 and TBLR1and Wnt target genes [210].

## Acetylation

Protein acetylation is a process that an acetyl group is transferred to the ϵ-amino group of an lysine residue of target protein [216]. Acetylation is catalyzed by acetyltransferase and the reverse process known as deacetylation is catalyzed by deacetylase [217,218]. Acetylation is well known to occur on N-terminal tail of histone protein to weaken DNA-histone interaction by neutralizing its positive charge, thereby activate gene expression [219-221]. Site-specific acetylation of a growing number of non-histone proteins involved in the regulation of diverse cell functions has been shown to regulate their activity, localization, specific interactions, and stability/degradation [222,223]. Using high-resolution mass spectrometry approach, an earlier study has identified 3600 lysine acetylation sites on 1750 proteins, suggesting that this modification is one of the most abundant chemical modifications in nature [224]. Nevertheless, to date, only a few components of the Wnt/β-catenin pathway including β-catenin and TCF are found regulated by acetylation. β-catenin is acetylated by the CREB-binding protein (CBP) acetyltransferase at lysine 49, a lysine site that is frequently found mutated in cancer [102]. Mutation of K49 activates transcription of Wnt pathway target in a promoter-specific fashion, implying a negative role of acetylation of β-catenin at K49 in Wnt signaling. In contrast, a later study shows that the transcriptional coactivator p300 upregulates β-catenin-dependent gene transcription by acetylating β-catenin at lysine 345, which increases the affinity of β-catenin for Tcf4 [103]. Consistent with this, a more recent study indicates that acetylation of β-catenin at lysine 19 and 49 by p300/CBP-associated factor (PCAF) stabilizes β-catenin, induces β-catenin nuclear translocation and increases its transcriptional activity, thereby upregulating Wnt signaling [104]. The positive role of β-catenin acetylation is further supported by a study showing that the NAD-dependent deacetylase sirt1 deacetylates β-catenin, leading to inhibition of β-catenin transcriptional activity and cell proliferation [225]. Identification of sirt1 deacetylation sites on β-catenin will help to elucidate the underlying mechanism by which acetylation of particular sites on β-catenin regulates Wnt signaling. Nonetheless, the observation that sirt1expression inversely correlates with nuclear β-catenin in human colon tumor specimens suggest the importance of balanced acetylation status of β-catenin in human disease.

Members of the TCF/LEF family are also substrates of acetylation [114-116]. It has been shown that *drosophila* CREB-binding protein (dCBP) binds to dTCF, acetylates lysine 25 in the Armadillo (β-catenin in *drosophila*)-binding domain of dTCF, which in turn lowers the affinity of Armadillo binding to dTCF, thereby repressing TCF [114]. In contrast, acetylation of the Caenorhabditis elegans LEF/TCF homolog POP-1 by CBP/p300 at lysines 185, 187 and 188 is required for POP-1 nuclear localization and biological activity during C. elegans embryogenesis [115], thus validating the physiological relevance of acetylation of POP-1, though POP-1 is considered to participate in the non-canonical Wnt pathway. The positive role of TCF acetylation in Wnt signaling is further supported by a recent study showing that human TCF4E2 can be acetylated at lysine 150 by CBP, leading to relief of transcriptional repression by transcription repressor presumably by inducing conformational change of TCF::DNA complex [116].

## ADP-ribosylation

ADP-ribosylation, which refers to the enzymatic transfer of one or more ADP-ribose from NAD + to the acceptor proteins [226], has been recognized as an important regulator in a wide range of biological processes, including DNA damage responses, transcriptional regulation, cell death as well as energy metabolism [227,228]. The importance of ADP-ribosylation in Wnt pathway is illustrated by the study in which TNKS1 and TNKS2 are identified to interact with Axin through its tankyrase-binding domain (a small amino-terminal region of axin 1, amino acids 19–30) and catalyze its poly-ADP-ribosylation, which in turn facilitates poly-ubiquitination and subsequent proteasome degradation of Axin [49]. Targeting TNKS by small molecule inhibitors including XAV939 [49] and WIKI4 [229] inhibits Wnt signaling and suppresses the malignant phenotypes including anchorage-independent growth in colorectal cancer cells, suggesting that TNKS is a potential target for treatment of Wnt-dependent cancers. More recently, in a screen to identify targets of ADP-ribosyltransferases ARTD10 and ARTD8, GSK3β is found to be modified by mono-ADP-ribosylation [62]. Further analysis indicates that this modification inhibits GSK3β activity *in vitro*. It is of great interest to understand the role of ADP-ribosylation of GSK3β in Wnt signaling in the future.

## Cross-talk between posttranslational modifications

PTMs often interplay to work in concert [21]. The interconnected modifications create multiple layer of regulation to fine tune the function of target protein and to determine the functional read-out. Crosstalk between PTMs has been classified as positive type or negative type [230]. Examples of positive crosstalk include priming phosphorylation, phosphorylation-dependent ubiquitination or sumoylation, and sumoylation-dependent ubiquitination. In these events, one PTM is believed to promote the addition of the second PTM through creating a binding site or recognition motif for the protein that is essential for the second PTM. The well-known example of negative crosstalk between PTMs is the competitive modification of different modifiers on a single residue.

Although crosstalk between PTMs has emerging as an important regulatory mechanism in signal transduction [21,231-233], most studies on the regulation of Wnt pathway by PTMs have focused on signal modifications. The best studied PTM crosstalk in Wnt/β-catenin pathway is the positive and negative interplay between PTMs on β-catenin: the sequential phosphorylation and ubiquitination of β-catenin [12,14,123] and competition between acetylation and ubiquitination of overlapping lysine residues on β-catenin [104]. In the case of positive interplay, β-catenin is phosphorylated at Ser45 by CK1α, which primes β-catenin for subsequent phosphorylation at Ser33, Ser37 and Thr41 by GSK3 and then triggers the recruitment of β-TrCP to β-catenin, resulting in poly-ubiquitination and degradation of β-catenin [12,14,123]. Lysines 19 and 49 on β-catenin are β-TrCP-dependent ubiquitination sites [93]. In the negative interplay case, acetylation of β-catenin at K19 and 49 by PCAF blocks β-catenin ubiquitination thereby stabilizing β-catenin [104]. How these three modifications are coordinated to control β-catenin protein level and the activation of the Wnt pathway in biological and pathological processes remains to be determined.

Another example of PTM crosstalk in the Wnt/β-catenin pathway is the poly-ADP-ribosylation-mediated polyubiquitination of Axin [49]. A recent study combining crystallographic and biochemical analysis sheds light on the mechanism of interplay between the two PTMs of Axin [234]. RNF146, The ubiquitin E3 ligase for Axin, contains a WWE domain and a RING domain, and is the only known E3 ubiquitin ligase to date that requires poly-ADP-ribosylation of the substrate for subsequent polyubiquitination [44,45,234,235]. The WWE domain of RNF146 specifically binds to iso-ADP-ribose (iso-ADPR), the smallest internal PAR structural unit containing the characteristic ribose–ribose glycosidic bond formed during poly-ADP-ribosylation rather than mono (ADP-ribose), leading to ubiquitination of poly-ADP-ribosylated Axin [234]. Several residues in RNF146 WWE domain are identified to be critical for iso-ADPR binding; sequence alignment further indicates these residues are conserved among WWE domains. Given that many ubiquitin E3 ligases contain WWE domain, it highly possible that poly-ADP-ribosylation is a general mechanisms to target protein for ubiquitination [234].

## Conclusions

Biological and clinical study over the past few years has greatly expanded our understanding of the complex Wnt signaling network, but there are still many fundamental aspects of the Wnt-related biology to be discovered and understood. Characterization, dynamic detection and quantification of chemical modifications of the pathway components are essential for us to gain deeper insight into biological control of the pathway. Although identification of PTMs involved in the Wnt/β-catenin pathway and validation of their function are far from completion—in fact a large number of PTMs are not all validated as physiological relevant, the concept that relaying Wnt signal requires dynamic changes in PTM states of the pathway components has been emerged. We now know that instead of relying solely on one particular modification, the Wnt pathway is controlled by the coordinated actions of phosphorylation, ubiquitination and other PTMs. However, little is known as to how PTM coordination is effectively achieved at the molecular and cellular level. For example, systematic analysis of PTM changes with respect to space and time remains an important future goal for PTM research in the Wnt field, and how signaling pathways or upstream enzymes that catalyze these PTMs interact combinatorially, hierarchically or reciprocally to ensure the sequence of the occurrence of the PTMs and kinetics of their durations during both “Wnt on and Wnt off” situations remains largely unexplored.

As the importance of PTMs has increasingly been appreciated, there is an increasing need for improved technologies that enable researchers to measure the state and function of PTMs in physiological situations. Characterizing the function of PTM is technically challenging due to the dynamic and reversible nature of PTM, and the complicated interplays between PTMs. A research trend in the field of PTM is the mouse knockin technology. Knockin methodology, coupled with gene knockouts and specific pharmacological inhibitors, is a powerful approach to dissect the physiological roles of individual modification at given sties on a given protein. However, it is an imperfect model because it knocks the protein permanently in one form—the protein with mimicked modification or the protein lacking modification, therefore it cannot recapitulate the dynamic and reversible nature of the PTMs.

Aberrant activation of Wnt/beta-catenin pathway contributes to development of different human cancers, especially colorectal cancers. As pointed by Nusse and Varmus [236], “among the most significant challenges in future research in the Wnt field is the identification of effective and specific Wnt pathway inhibitors for use in cancer and other diseases”. Unfortunately, Wnt signaling pathway is difficult to target [237,238]. The success of tankyrases inhibitors –XAV939 [49] and WIKI4 [229]— as Wnt pathway inhibitors by inhibiting poly-ADP-ribosylation of Axin suggests that modulating PTMs of the Wnt pathway components represents a promising alternative approach for targeting the Wnt pathway. To this end, we believe that a better understanding of the regulation of the Wnt/β-catenin pathway by PTMs could have far-reaching implications for identifying novel approaches for targeting Wnt signaling.

## Competing interests

The authors declare that they have no competing interests.

## Authors’ contributions

CG, GX, and JH wrote the review. All authors read and approved the final manuscript.
